# On the Mechanism of Action of SJ-172550 in Inhibiting the Interaction of MDM4 and p53

**DOI:** 10.1371/journal.pone.0037518

**Published:** 2012-06-04

**Authors:** Michal Bista, David Smithson, Aleksandra Pecak, Gabriella Salinas, Katarzyna Pustelny, Jaeki Min, Artur Pirog, Kristin Finch, Michal Zdzalik, Brett Waddell, Benedykt Wladyka, Sylwia Kedracka-Krok, Michael A. Dyer, Grzegorz Dubin, R. Kiplin Guy

**Affiliations:** 1 Max-Planck Institute for Biochemistry, Martinsried, Germany; 2 Department of Analytical Biochemistry, Faculty of Biochemistry, Biophysics and Biotechnology, Jagiellonian University, Krakow, Poland; 3 Department of Chemical Biology and Therapeutics, St. Jude Children’s Research Hospital, Memphis, Tennessee, United States of America; 4 Department of Physical Biochemistry, Faculty of Biochemistry, Biophysics and Biotechnology, Jagiellonian University, Krakow, Poland; 5 Department of Developmental Neurobiology, St. Jude Children’s Research Hospital, Memphis, Tennessee, United States of America; 6 Department of Microbiology, Faculty of Biochemistry, Biophysics and Biotechnology, Jagiellonian University, Krakow, Poland; 7 Hartwell Center for Biotechnology, St. Jude Children’s Research Hospital, Memphis, Tennessee, United States of America; 8 Malopolska Centre of Biotechnology, Krakow, Poland; 9 Department of Ophthalmology, University of Tennessee Health Science Center, Memphis, Tennessee, United States of America; 10 Howard Hughes Medical Institute, Chevy Chase, Maryland, United States of America; Stanford University, United States of America

## Abstract

SJ-172550 (**1**) was previously discovered in a biochemical high throughput screen for inhibitors of the interaction of MDMX and p53 and characterized as a reversible inhibitor (J. Biol. Chem. 2010; *285*∶10786). Further study of the biochemical mode of action of **1** has shown that it acts through a complicated mechanism in which the compound forms a covalent but reversible complex with MDMX and locks MDMX into a conformation that is unable to bind p53. The relative stability of this complex is influenced by many factors including the reducing potential of the media, the presence of aggregates, and other factors that influence the conformational stability of the protein. This complex mechanism of action hinders the further development of compound **1** as a selective MDMX inhibitor.

## Introduction

The p53 pathway is inactivated in virtually every human cancer by mutations in the p53 gene itself or other genes in the pathway [1]. One of the most common mechanisms of p53 suppression in tumors with wild type p53 is increased expression of the p53 antagonists MDM2 or MDMX (MDM4) [2], [3]. In some tumors, the increased expression of MDM2 or MDMX correlates with genetic amplification, but there are also other non-genetic mechanisms that can contribute to increased protein levels such as alternative splicing [4] or changes in miRNA-mediated regulation of steady state mRNA and protein levels [5]. For tumors with elevated levels of MDM2 or MDMX and wild type p53, it may be possible to induce p53-mediated cell death by disrupting the MDM2-p53 or MDMX-p53 interaction. Indeed, Vassilev and colleagues [6] identified the first small molecule inhibitor of MDM2-p53 called nutlin-3a and showed that this MDM2 antagonist can induce cell death in cancer cells with elevated MDM2 in a p53 dependent manner. Since the original report of nutlin-3a, several other MDM2 antagonists have been reported. [7]–[9].

**Figure 1:**
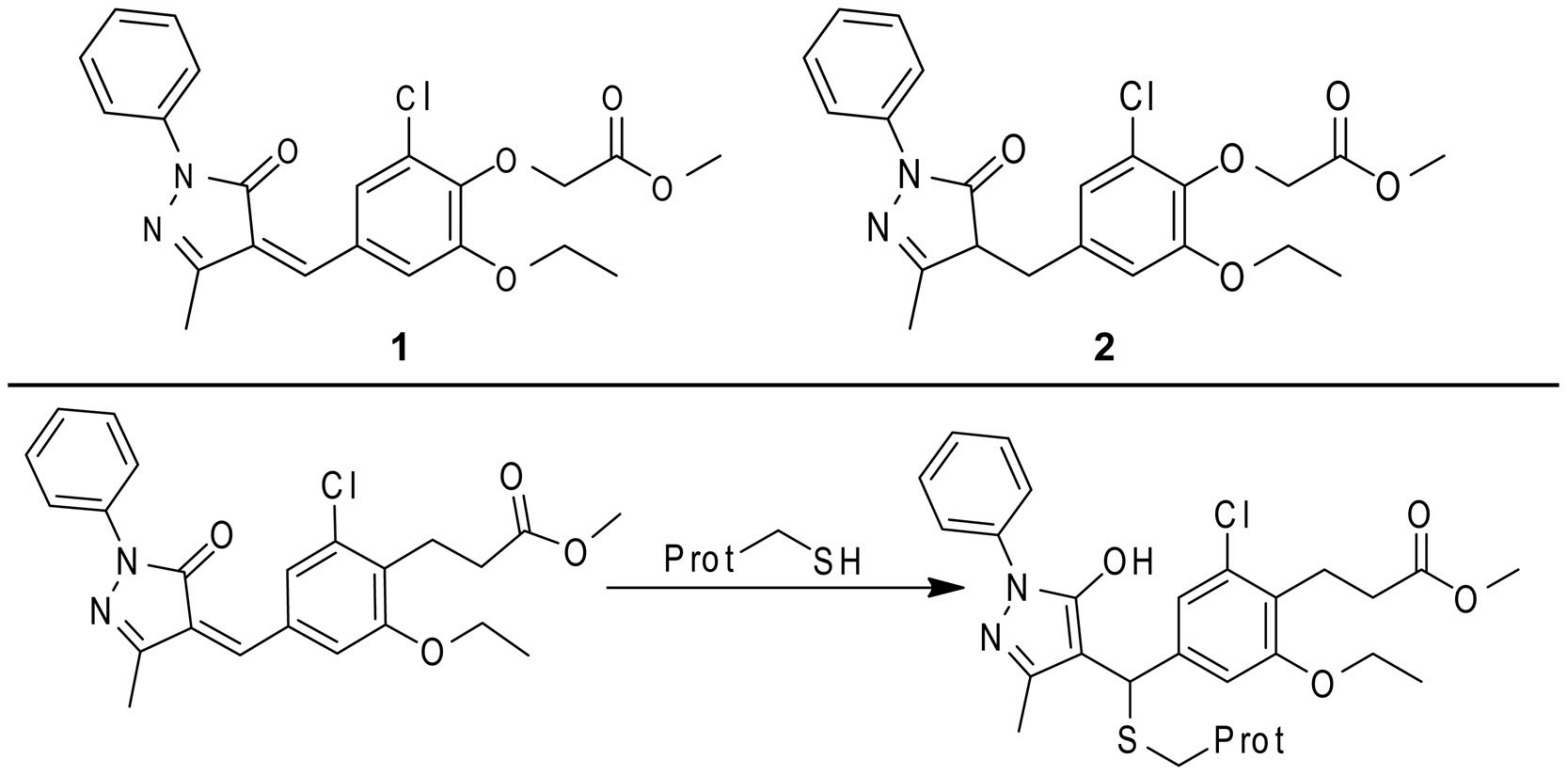
Structures of relevant compounds. **Panel A.** Structure of SJ-172550 (**1**) and a non-reactive analog (**2**). **Panel B.** The potential mechanism of covalent adduct formation.

More recently, small molecule inhibitors of MDMX have been described. The first (Figure 1, Panel A, SJ-172550, **1**) was identified in a high-throughput screen using a biochemical assay to recapitulate the binding of MDMX and p53 [10]. SJ-172550 could compete for the wild type p53 peptide binding to MDMX with an EC_50_ ∼ 5 µM and caused p53 dependent cell death of retinoblastoma cells [10]. For comparison, nutlin-3a inhibited the MDMX-p53 interaction with an EC_50_ ∼ 30 µM [10]. Another small molecule inhibitor of MDMX-p53 that has been characterized is WK298 [11]. This molecule binds to MDMX with a binding constant of ∼ 20 µM and structural studies have shown that it mimics binding of the p53 peptide [11]. In addition to small molecule inhibitors of the MDMX-p53 interaction, there have been several reports of peptide inhibitors [12]. For example, a stapled peptide that mimics the p53 helix that interacts with the MDMX protein was effective at disrupting the MDMX-p53 interaction *in vitro* and *in vivo* [13].

The chemotype embodied by compound **1** contains a functional group, an α,β-unsaturated amide, that is capable of undergoing reaction with protein sulfhydryls to form covalent adducts (**Figure 1** **, Panel B**). Indeed compound **1** will form adducts with glutathione (**Figure S1)** or cysteine containing peptides (**Figure S2)** under forcing conditions. While irreversible inhibitors remain a viable option for clinical development, particularly in oncology, [14]–[17] they carry certain liabilities as a class and require a high degree of selectivity either at the protein level or the site of action, or ideally both. In order to properly delineate the risk involved in developing an irreversible inhibitor it is critical to understand its mechanism of action at the protein level and how this will relate to efficacy on the target, pharmacokinetics, and off-target effects.

In prior work, we carried out preliminary characterization of the mechanism of action of **1** using two experiments: mass spectrometry of MDMX treated with **1**; and a washout of **1** from treated MDMX, followed by a functional test of recovered protein. [10] In the first case, incubation of MDMX with **1** for two hours in non-reducing but otherwise pseudo-physiological buffer, followed by dialysis, afforded no detectable covalent adducts by MALDI mass spectrometry. Likewise, when this protein was tested for its ability to bind p53 peptide, it retained function. The interpretation of these results was that **1** acted as a reversible inhibitor of the interaction of p53 and MDMX. However, two subsequent findings led us to explore this conclusion more fully: 1) Compound **1** only appeared to function under non-reducing conditions; and 2) allowing MDMX and compound **1** to react in neutral pH acetate buffer gave clear covalent adducts detectable by mass spectrometry (see Figures S7, S8, S9, and S10).

Therefore, we carefully analyzed the molecular mechanism of action. These studies led to three major findings: 1) MDMX exists as an ensemble of conformations that differ in both their ability to bind p53 and their reactivity with alkylating agents; 2) in non-reducing conditions or high inhibitor and MDMX concentrations, the majority of MDMX adopts a conformation that fails to bind p53 and is sensitive to alkylation - under these conditions **1** binds covalently but reversibly to MDMX; and 3) under reducing conditions the majority of MDMX adopts a conformation that is competent to bind p53 and relatively non-reactive with compound **1**. Additionally, compound **1** appears to shift the MDMX conformational equilibrium towards the conformation unable to bind p53 by reversible alkylation of Cys76. This complex, multi-mode mechanism greatly complicates the interpretation of experiments using **1** and limits its value as a lead compound for further development as a selective MDMX inhibitor.

## Results and Discussion

### Demonstration of the Formation of Covalent Adducts between Compound 1 and MDMX

**Figure 2:**
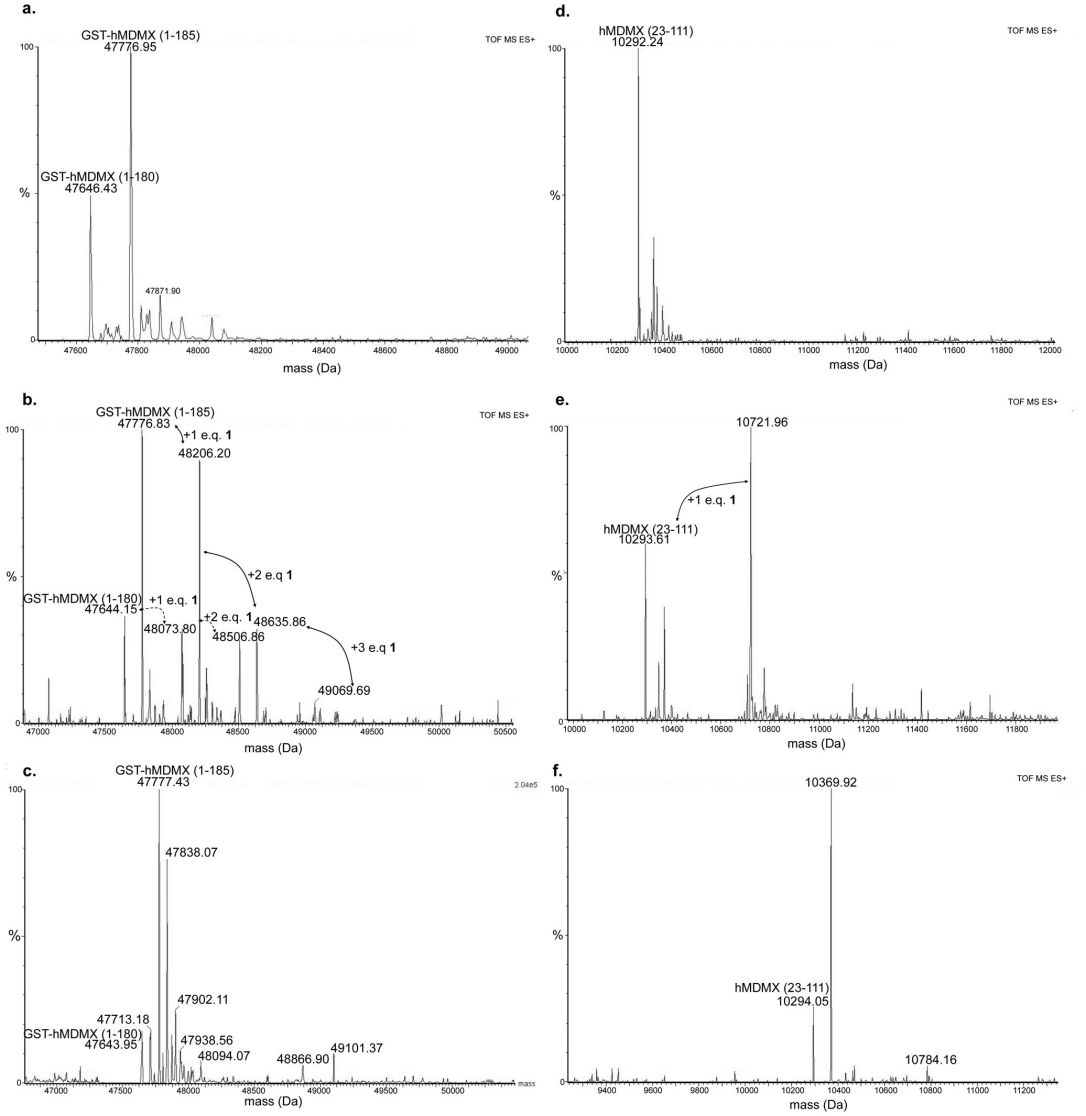
Formation of covalent adducts between compound 1 and MDMX. **Panel a.** Mass spectrum arising from unmodified hMDMX (GST-tagged screening construct) showing unmodified mass of the protein. **Panel b.** Mass spectrum arising from treatment of 20 µM GST-hMDMX with 100 µM of compound **1** demonstrating multiple alkylation events. Note that 100 µM is well above the solubility limit of compound **1** and significant aggregation of compound exists. **Panel c.** Mass spectrum arising from treatment of 1 µM GST-hMDMX with 5 µM of compound **1** demonstrating no alkylation events. **Panel d.** Mass spectrum arising from unmodified hMDMX (untagged aa 23 to 111 construct) showing unmodified mass of the protein. **Panel e.** Mass spectrum arising from treatment of 20 µM hMDMX with 100 µM of compound **1** demonstrating partial alkylation. **Panel f.** Mass spectrum arising from treatment of 1 µM hMDMX with 5 µM of compound **1** demonstrating no alkylation.

In our original report, mass spectrometry following dialysis of a mixture of compound **1** and MDMX failed to yield detectable adducts [10]. However, preliminary mass spectrometry experiments involving direct injection of a mixture of compound **1** and MDMX revealed clear adduct peaks (**Figures S7, S8, S9, and S10)**. This disparity prompted us to more carefully study the mechanism of action of compound **1**. We hypothesized that the difference between the two experimental results might be explained by reversibility of the adduct formation over the time course of tens of minutes combined with the use of extended dialysis in prior experiments to remove excess compound **1**. Therefore, we undertook studies to examine the formation of adducts directly by mass spectrometry of mixtures of MDMX and compound **1** without prior separation by dialysis (**Figure 2**). In order to eliminate the possibility that adducts were forming solely on the GST fusion tag (because tagged protein was used for all prior experiments) the experiments were carried out side-by-side with protein containing or not containing the tag. In both cases, the unmodified protein ionized well from the buffer mixture and the expected correct mass could be detected (**Figure 2** **, Panels a and d**). When high concentrations of compound **1** were allowed to react with either construct of the protein (20 µM protein; 100 µM compound **1**), and injected with minimal manipulation, adducts clearly formed, with either multiple alkylation events (GST-tagged protein) or a single alkylation event (untagged protein). These constructs were stable enough to at least partially survive a rapid desalting procedure prior to injection in the mass spectrometer. In the case of the untagged protein this stoichiometry afforded only partial labeling, which may reflect either low reactivity, or rapid reversal during desalting, or both. Thus, clearly compound **1** is capable of alkylating the single cysteine present in the p53 binding domain of MDMX. However, under these conditions the compound is not fully soluble (solubility of 12 µM in aqueous buffer) and exists mostly in aggregated form, which we have previously shown can result in aberrant protein behavior. [18].

In order to test whether aggregation was a driver for the observed activity of compound **1**, the reactions were also carried out at conditions more closely resembling those used in the original report (20-fold reduction in total concentration of each component, same stoichiometry). Under these conditions the binding of p53 peptide to MDMX is fully inhibited at equilibrium. In order to provide enough protein to give strong signal in the mass spectrometer, this mixture had to be rapidly concentrated (5 minutes) prior to desalting. No adducts were detected for either protein construct. One can conclude from these experiments and the data published in our original report [10] that the interaction of compound **1** with MDMX is covalent but reversible.

These experiments also suggest that any conditions that perturb conformational equilibria may lead to adduct formation. We have carried out similar experiments with a wide variety of protein constructs of hMDMX and hMDM2 and demonstrated that covalent adduct formation can occur with compound **1** independent of construct and in a wide variety of conditions (Figures S7, S8, S9, and S10). Adduct formation is enhanced by changes in ionic concentration, substantial increases in protein concentration, or use of large amounts of the inhibitor (higher than its solubility limit). The adduct formation appears to require the cysteine residue within the protein.

### Molecular Mechanism of Interaction of Compound 1 and MDM Family Members

**Figure 3:**
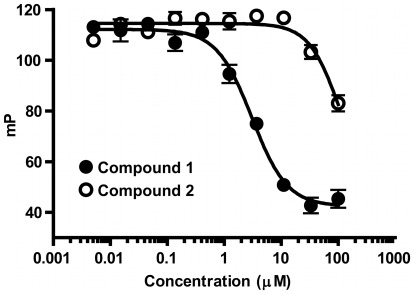
Inhibition of MDMX-p53 peptide binding by compound 1 (IC_50_ = 3 µM), compound 2 (IC_50_>100 uM).

Careful consideration of these results suggests that compound **1** exerts its effect upon MDMX binding p53 by forming a covalent complex with MDMX through reaction with the cysteine residue in the binding domain but that the resulting adduct exists in dynamic equilibria rather than being irreversible. If this were the case, then the activity would be dependent upon the presence of the electrophilic group within the chemotype and upon the presence of the cysteine. To test this hypothesis, an experiment was carried out to compare inhibition of MDMX-p53 peptide binding with compound **1** and compound **2**, which lacks the required electrophilic center (**Figure 3**). Saturation of the ene-amide group reduced the inhibitory potency by at roughly 30-fold, consistent with covalent bond formation being important to the mechanism of action. A similar result could be obtained by allowing compound **1** to react with glutathione *in situ*, reducing potency by 10-fold but not completely removing efficacy (**Figure S5**). These experiments suggest that the binding of compound **1** to MDMX utilizes both covalent and non-covalent interactions. Most likely this takes the form of pre-organization of the compound with the “active site” cysteine, followed by formation of a covalent adduct, which we have previously reported with other cysteine reactive electrophilic inhibitors [19].

The hypothesis also implies that the interaction requires the presence of the cysteine residue in the binding domain. While we have not tested this hypothesis on MDMX, we have shown that a mutant of MDM2 protein (hMDM2(18–125)C77V) lacking the cysteine (**Figure S6**) is inhibited by compound **1** with 10-fold weaker potency than that seen with the MDM2 while the p53 binding potency of the mutant remains the same as that of the wild type MDM2. This reinforces the key concept outlined above – the interaction between compound **1** and MDMX/2 involves both covalent and non-covalent components with both being required for maximal potency. Moreover, the covalent component requires the presence of both the unsaturated functionality of compound **1** and the cysteine of the protein. The most likely mechanism is outlined in **Figure 1**.

### Conformational Flexibility of MDMX

MDMX contains a single cysteine residue within the N-terminal p53 binding domain. Crystallographic studies have shown that the residue is buried within the protein (inaccessible to the solvent). Clearly, covalent modification would require at least partial unfolding of the protein. The initial hypothesis concerning reconciling the variation in alkylation with **1** was that the varying buffer conditions were leading to partial denaturation of the MDMX protein rendering it non-functional and simultaneously revealing the cysteine - thus allowing for reaction with the unsaturated group on **1**. In order to test this concept, we carried out two series of experiments: 1) examination of binding of MDMX immobilized to a surface by p53 peptide using surface plasmon resonance (SPR) and 2) examination of the thermal stability of MDMX.

**Figure 4:**
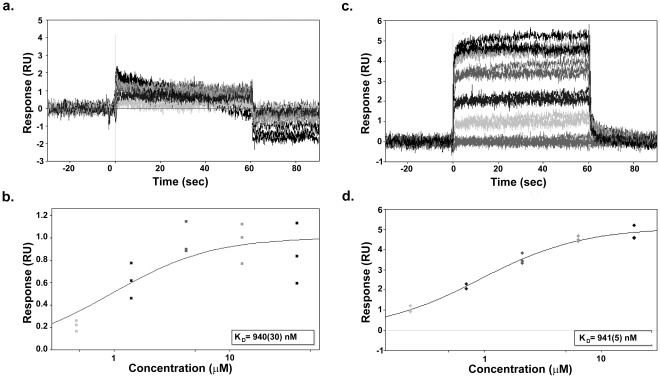
Variation in function of MDMX depending upon buffer conditions. **Panel** **a.** SPR binding of hMDMX(23–111) to p53 peptide under non-reducing conditions. Note poor response indicating many of the protein molecules are not “active.” **Panel** **b.** The p53 binding curve generated in non-reducing conditions showing a K_d_ of 940 nM. **Panel** **c.** SPR binding of hMDMX(23–111) to p53 in the presence of 1 mM TCEP is much improved, indicating that more of the protein molecules are “active.” **Panel** **d.** The p53 binding curve generated under reducing conditions – the K_d_ is the same as that determined under non-reducing conditions.

The results of the SPR study are shown in **Figure 4**. In these experiments, hMDMX (23–111) was immobilized to the SPR chips using a biotin tag. Synthetic p53 peptide, in the presence or absence of reducing agent, was then flowed across the SPR chip and binding measured. When the experiment is carried out under non-reducing conditions (**Figure 4** **, Panels a and b;** mimicking the conditions of the original assays), the p53 peptide does bind, but magnitude of the response is small (**Panel a**), the data quite noisy, and the resulting binding isotherm not well defined (**Panel b**). On the other hand, when the same protein on the same chip is reduced *in situ* (1 mM TCEP) and probed with p53 peptide, the response is robust (**Panel c**), the data clean, and the resulting binding isotherm well defined (**Panel d**). Of note is the fact that the measured K_d_ remains the same for both assays.

Thus, it appears that a substantial proportion of the protein in the non-reducing conditions is not functionally active. However, this is not a case of classic denaturation but rather a partial unfolding because the protein remains dis-aggregated on analytical chromatography (data not shown) and regains function with addition of reducing agents. The comparable K_d_ values suggest that the conformer responsible for binding p53 remains the same in both conditions while reversibility of the “inactive” state strongly suggests that the differences in behavior are due to changes in conformer populations.

**Figure 5:**
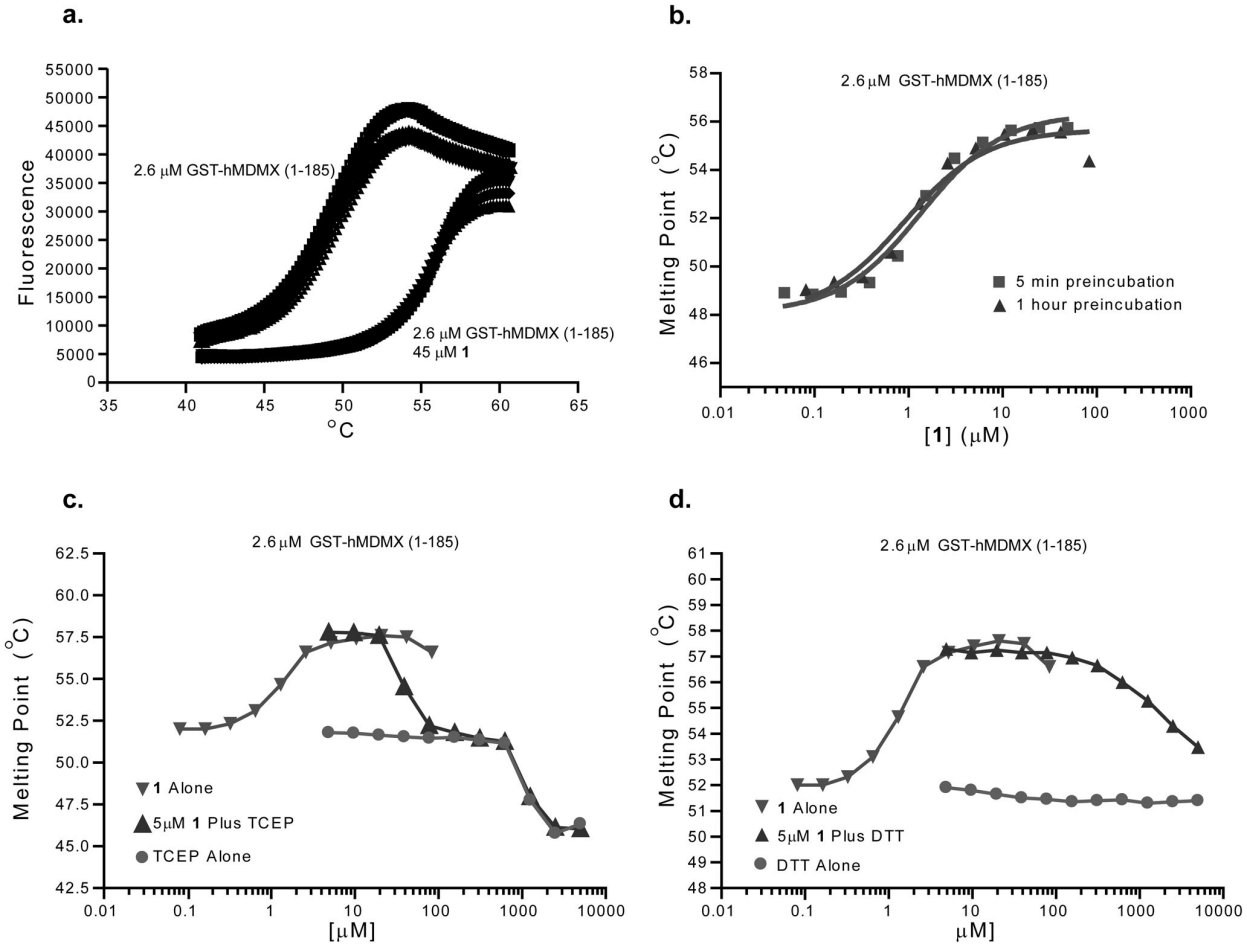
Thermal stability equilibria of MDMX. **Panel a.** Thermal shift data for MDMX (23–111) showing a 7 degree stabilization of the protein’s melting point by addition of compound **1**. The panel shows individual data sampling points from 3 independent experiments from each condition. **Panel b.** Dose dependency and time dependency of the effect showing an apparent EC_50_ of roughly 1 µM and minimal time dependency. **Panel c.** Dose dependent reversal of the effects of compound **1** by TCEP. **Panel d.** Dose dependent reversal of the effects of compound **1** by DTT.

Next, the effects of buffer condition changes and exposure to compound **1** upon the conformational equilibria of MDMX were examined by a different technique – thermal stability as measured by hydrophobic dye binding (**Figure 5**) [20]. Initially, MDMX was allowed to interact with varying concentrations of **1** for 1 h. Then the dye binding capacity of the protein was assessed across a temperature range in order to induce a phase transition from low to high dye binding – normally interpreted as the “melting point” of the protein – the point at which the conformational flexibility of the protein cooperatively opens to many confirmations (**Panel a)** [9]. In this case, compound **1** increases the temperature required to reach a phase transition, which would normally be interpreted as increasing stability. Our prior work has shown that similar covalent inhibitors of protein interactions often show slow on rates, relative to non-covalent inhibitors, and will show time dependencies in their behaviors. [21]–[23] In order to assess if the shift in MDMX melting point was time dependent the experiment was carried out with long (1 h) and short (5 min) incubation times; no change was observed in the phase transition temperature. Next, the effects of reducing agents were examined. For both TCEP and DTT, addition of the reducing agent to the preformed mixture of MDMX and compound **1** (at apparent EC50 from the first experiment) reversed the stabilization of the protein caused by compound **1**. When used alone, TCEP actually destabilized the protein at high concentrations while DTT had no apparent effect. This study strongly suggests that the binding of **1** to MDMX is reversible and that its effect is suppressed by reducing agents, whether or not they contain a nucleophilic thiol.

### The Nature of the Reversibility of the Binding of Compound 1 to MDMX

Key issues arising from these studies are whether or not the interaction between compound **1** and the cysteine residue is truly as depicted in Figure 1 (Panel b) and how it may influence or be influenced by the conformational equilibrium of MDMX described above. Preliminary experiments indicated that **1** could form stable adducts with glutathione and with cysteine containing peptides as detected by LC/MS (**Figures S1 & S2**). This was also true with other analogs (**Figure S11 and S12)** that bind MDM2 and MDMX (**Table S1).** This raises the possibility that the reversion of inhibition after treatment with reducing agents is due to the trapping of compound **1** by excess nucleophilic reducing agent while at equilibrium. Additionally, if MDMX was treated with Ellman’s reagent (DTNB), which is known to form mixed dithianes, the protein became unable to bind p53 peptide and the melting point was partially stabilized (**Figures S3 and S4**). This raises the possibility that DTT, which is capable of forming such species might reverse the effects of compound **1** by inducing formation of a new protein adduct.

**Figure 6:**
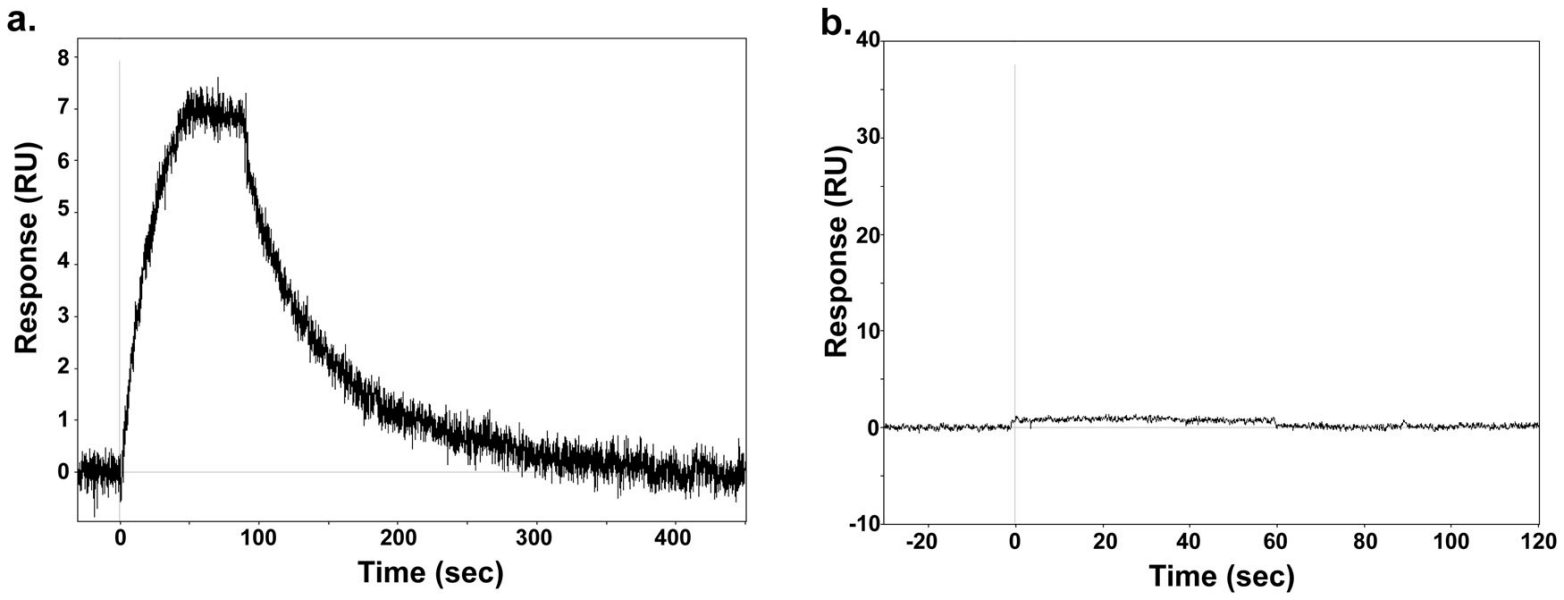
Reversibility of the interaction of compound 1 with MDMX. **Panel a.** SPR study of the binding of **1** (100 µM) to hMDMX (aa 23–111) under non-reducing conditions. While the off-rate is slow, the interaction is reversible. **Panel b.** SPR study of the binding of **1** (100 µM) to hMDMX (aa 23–111) under reducing conditions. No binding is observed.

In order to address these issues, compound **1** was allowed to interact with MDMX in the presence or absence of TCEP (a non-thiol reducing agent) and binding monitored by SPR. TCEP is neither expected to trap compound **1** nor form adducts with MDMX. When MDMX was immobilized to the chip and then treated with compound **1** in the absence of reducing agent, there was clear strong signal representing binding (**Figure 6** **, Panel a**) and after the initial binding a decay of signal indicating that compound **1** binds reversibly. The off rate was relatively slow, requiring almost 5 minutes to return to baseline after the pulse of compound **1**. If the same experiment was carried out in the presence of the non-nucleophilic reducing agent no binding was observed (**Panel b**). In control experiments where compound **1** was exposed to an excess of TCEP in the same buffers used for the MDMX protein experiments, no adduct formed between compound **1** and TCEP (data not shown). While not unexpected, this clearly indicates that compound **1** should be available for covalent reaction with the reduced cysteine. This study indicates that the binding of **1** to MDMX is reversible and requires access to a conformation that is suppressed by reducing conditions. This also explains why no covalent adduct is observed after treatment of MDMX with **1** when the protein is dialyzed prior to mass spectrometry – the dialysis time was sufficient to allow reversal of the reaction.

### Model of Interaction of MDMX with 1

The studies described above suggest a consistent model of MDMX inhibition by compound **1**. MDMX exists in a dynamic conformational equilibrium of at least two states that is biased both by the effects of compound **1** and reducing agents. In one state, which is favored by non-reducing conditions and concentrations of compound **1** above the solubility limit, MDMX is not competent to bind p53 and the side chain of cysteine 76 is exposed to the solvent and thus available for alkylation. Alkylation of Cys76 with **1** stabilizes this form, thus explaining the effect of compound **1** on p53 binding. A second state is favored in the presence of reducing agents and inhibitor concentrations below the solubility limit. In this state MDMX is able to bind p53 and the cysteine 76 side chain is buried inside the protein and thus resistant to alkylation. The fact that irreversible alkylation with DTNB also blocks p53 binding suggests that any alkylation of cysteine 76 will result in a conformational bias towards the conformation unable to bind p53. The mechanism by which reducing agents push the equilibrium towards the p53 binding competent conformation is unclear and may not simply be explained by forcing Cys76 into a reduced state.

Thus, the MDMX protein exists in a complex conformational equilibrium that is biased both by the effects of compound **1** and reducing agents. Taken together these studies demonstrate that MDMX exists in conformers that are respectively either 1) relatively conformationally closed and competent to bind p53 peptide; or 2) relatively conformationally open and incompetent to bind p53 peptide. These conformational states appear to be in equilibrium with one another. Compound **1** appears to lock the protein into the latter state while reducing agents push it towards the former. These findings highlight the complex mechanism of compound **1,** which does not appear to act as a simple competitive inhibitor but rather by affecting the conformation of its target protein. The study also emphasizes the role of the single cysteine residue in the p53 binding domain of MDMX which appears to be critical both for controlling the conformational equilibria of the protein and for the mechanism of action of compound **1.** The originally reported data on reversibility of **1** were thus only a part of the full story uncovered here. This complex, multi-mode mechanism complicates the interpretation of experiments using **1** and limits its value as a lead compound for further development as a selective MDMX inhibitor.

## Materials and Methods

### Protein Expression and Purification

A GST fusion protein of the p53-binding domain of human MDMX (a.a. 1–185) was cloned into a pGEX-4T1 plasmid. A second construct expressing a shorter hMDMX (a.a. 23–111) with a HIS-tag into a pGEX plasmid. Both constructs were expressed in BL21(DE3) *E. coli*. The bacterial cells were collected by centrifugation and lysed by sonication. Lysates were cleared by ultra centrifugation (100,000 g for 30 minutes). For the GST-tagged version the supernatant was purified using a GSTrap Fast-Flow column (GE Healthcare). For the His-tagged version, the protein was purified using Talon nickel resin and the His-tag was subsequently removed by proteolytic cleavage. In both cases, the eluted protein was further purified by a Mono Q anion-exchange chromatography and gel filtration using S200 column. Peak fractions were combined and dialyzed into phosphate-buffered saline (pH 7.6) with 2 mM phenylmethylsulfonyl fluoride. Biotin-MDM4 was prepared using avi-tagged MDM4 (a.a. 23–111) and biotin labeled as recommended by the vendor (Genecopeia).

### Isothermal Denaturation (ITD)

The ITD measurements were performed with a RT-PCR instrument (Applied Biosystems 7900HT) equipped with a 384-well probe using SYBR detection (excitation 495/emission 537). The program used started at 45°C and the temperature increased at a rate of 1 degree per minute. The final concentrations were 0.125 mg/ml of GST-hMDMX (a.a. 1–185), varying concentrations of test compound (25 nM to 100 µM) and Sypro orange (Invitrogen) at 5-fold the protein concentration in a buffer consisting of 10 mM TRIS (pH 8.0) and 25 mM NaCl [24]. The final volume was 20 µl for each well using 384 ABI PRISM™ plates (Applied Biosystems). The data was exported and evaluated using a custom data processing algorithm coded in Pipeline Pilot (Scitegic). Apparent melting temperatures were calculated by measuring the temperature at which the observed fluorescence signal was halfway between the baseline and the maximum. Binding curves were determined by graphing observed apparent melting temperature as a function of test compound concentrations. These curves were then fit to a four parameter sigmoidal dose response model using PRISM 5.0 (GraphPad Software, Inc.).

### Solubility

Solubility assay was carried out on Biomek FX lab automation workstation (Beckman Coulter, Inc., Fullerton, CA) using µSOL Evolution software (pION Inc., Woburn, MA). The detailed method is described as following. 10 µL of 10 mM compound stock (in DMSO) was added to 190 µL 1-propanol to make a reference stock plate. 5 µL from this reference stock plate was mixed with 70 µL 1-propanol and 75 µL SSB (system solution buffer, pH adjusted to 7.4, pION Inc., Woburn, MA) to make the reference plate, and the UV spectrum (250 nm –500 nm) of the reference plate was read. 6 µL of 10 mM test compound stock was added to 594 µL SSB in a 96-well storage plate and mixed. The storage plate was sealed and incubated at room temperature for 18 hours. The suspension was then filtered through a 96-well filter plate (pION Inc., Woburn, MA). 75 µL filtrate was mixed with 75 µL 1-propanol to make the sample plate, and the UV spectrum of the sample plate was read. Calculation was carried out by µSOL Evolution software based on the AUC (area under curve) of UV spectrum of the sample plate and the reference plate. All compounds were tested in triplicates.

### Fluorescence Polarization Assay

All fluorescence polarization assays were performed in binding buffer (10 mM Tris, pH 8.0, 170 mM NaCl, 0.05% Tween-20) unless otherwise noted. For competition assays a master mix of 15 nM Texas red-labeled (N-terminal label) P53 Peptide (sequence: GSGSSAETFSDLWKLLPEN) with 1 µM hMDMX (GST-tagged, a.a. 1–185) was prepared and 20 µl was added to black polystyrene 384 well plates (Corning #3573). DMSO stock solutions of test compounds were then added by pin transfer (V&P scientific) in nano-liter volumes (maximum final DMSO percentage  = 1%). The assay mixture was incubated at room temperature for one hour and read on an EnVision multi-label plate reader using a 555 nm excitation filter, a 632 nm emission filter, and a Texas Red FP dichroic mirror. Binding inhibition curves and IC_50_ values were determined by fitting observed mP values to a 4-parameter sigmoidal binding curve using PRISM 5.0 (GraphPad Software, Inc.).

### Mass Spectroscopy Experiments

Samples were prepared in binding buffer (10 mM Tris, pH 8.0, 170 mM NaCl) with a 50 µl final volume. Final concentration of test compounds (0 to 100 µM) and hMDMX constructs (1 or 20 µM) were varied as indicated in figure legends. Samples were incubated at room temperature for 1.5 hours after which they were stored overnight at 4°C before analysis by mass spectrometry. Samples containing 1 µM hMDMX constructs were concentrated to 20 µM using 3000 MWCO centrifugal protein concentrators (Vivascience, Inc.) immediately prior to analysis. Prior to injection all samples were desalted using a reverse phase C8 Zip Tip (Millipore) and eluted in 50% acetonitrile, 2% formic acid. The samples were ionized by static nanospray using EconoTips (New Objective) on a Waters LCT Premier XE mass spectrometer using positive mode. The resultant charge envelope was deconvoluted using MaxEnt 1 algorithm of MassLynx V 4.0 sp 4 software. A mass error of 1Da for every 10,000 Da is permissible using this mass spectrometer.

### Surface Plasmon Resonance Assay

Binding studies were performed at 25°C using a Biacore T100 (GE Healthcare) surface plasmon resonance (SPR) instrument. Either streptavidin or NeutrAvidin (Thermo Scientific) was immobilized on a carboxymethyldextran-coated gold surface (CM5 chip; GE Healthcare) by standard amine coupling methods. The carboxymethyl groups of dextran were activated with *N*-ethyl-*N* ´-(3-dimethylaminopropyl) carbodiimide (EDC) and *N*-hydroxysuccinimide (NHS). For peptide experiments, streptavidin was attached at pH 4.5 in 10 mM sodium acetate to levels of ∼3000 RU per flow cell. For small molecule experiments, NeutrAvidin was attached at pH 5.0 in 10 mM sodium acetate to levels of ∼11000–12000 RU per flow cell. Any remaining reactive sites were blocked by reaction with ethanolamine. Biotin-MDM4 was captured on the chip by injection over the streptavidin or NeutrAvidin surface.

For peptide binding experiments, p53wt peptide was prepared as a 3-fold serial dilution in peptide binding buffer (20 mM Bis-Tris pH 6.5, 200 mM NaCl, 0.1 mg/mL bovine serum albumin, 0.005% Tween20, +/−1 mM TCEP). The peptide was injected in triplicate at each concentration at a flow rate of 75 µL/min. In non-reducing conditions the peptide concentration range was 38 µM –469 nM, and in reducing conditions it was 19 µM –235 nM. For small molecule binding, compound SJ-172550 (**1**) was prepared in binding buffer (20 mM Bis-Tris pH 6.5, 200 mM NaCl, 0.01% Tween20, 5% DMSO) and injected at a single concentration of 100 µM at a flow rate of 100 µL/min. Data were processed, double-referenced and solvent corrected (where appropriate) using the software package Scrubber2 (version 2.0 b, BioLogic Software). Equilibrium dissociation constants (*K*_d_) for peptide binding were determined by equilibrium affinity analysis with a 1∶1 binding model using Scrubber2.

## Supporting Information

Figure S1 **Formation of adducts by glutathione and compound 1.**
(TIF)

Figure S2 **Formation of adducts by cysteine containing peptide and compound 1.**
(TIF)

Figure S3 **Inhibition of interaction of p53 and MDMX with DTNB.**
(TIF)

Figure S4 **Partial Stabilization of melting point of MDMX with DTNB.**
(TIF)

Figure S5 **Effects of glutathione upon potency of compound 1 and nutlin in blocking binding of p53 peptide by MDMX.**
(TIF)

Figure S6 **C77A mutation abolishes the ability of compound 1 to covalently label MDM2.**
(TIF)

Figure S7 **Formation of adducts by hMDMX(1–134) and compound 1.**
(TIF)

Figure S8 **Formation of adducts by hMDMX(1–111) and compound 1.**
(TIF)

Figure S9 **Formation of adducts by hMDM2(1–118) and compounds 1 and 3.**
(TIF)

Figure S10 **Formation of adducts by hMDM2(1–125) and compound 1.**
(TIF)

Figure S11 **Formation of adducts by β-mercaptoetanol and compound 3 evidenced by NMR.**
(TIF)

Figure S12 **Reduced sensitivity of C77A mutant of MDM2 to compound 3 but not nutlin in blocking the interaction with p53 peptide.**
(TIF)

Table S1 **Binding affinities of close and distant derivatives of 1 towards Mdm2 and Mdmx.**
(PDF)

## References

[1] Hanahan D, Weinberg RA (2011). Hallmarks of cancer: the next generation.. Cell.

[2] Vazquez A, Bond EE, Levine AJ, Bond GL (2008). The genetics of the p53 pathway, apoptosis and cancer therapy.. Nat Rev Drug Discov.

[3] Marine JC, Dyer MA, Jochemsen AG (2007). MDMX: from bench to bedside.. J Cell Sci.

[4] Mancini F, Di Conza G, Moretti F (2009). MDM4 (MDMX) and its Transcript Variants.. Curr Genomics.

[5] Wynendaele J, Bohnke A, Leucci E, Nielsen SJ, Lambertz I (2010). An illegitimate microRNA target site within the 3' UTR of MDM4 affects ovarian cancer progression and chemosensitivity.. Cancer Res.

[6] Vassilev LT, Vu BT, Graves B, Carvajal D, Podlaski F (2004). In vivo activation of the p53 pathway by small-molecule antagonists of MDM2.. Science.

[7] Herold JM, Wigle TJ, Norris JL, Lam R, Korboukh VK (2011). Small-molecule ligands of methyl-lysine binding proteins.. Journal of medicinal chemistry.

[8] Artz JD, Wernimont AK, Allali-Hassani A, Zhao Y, Amani M (2011). The Cryptosporidium parvum kinome.. BMC genomics.

[9] Senisterra G, Chau I, Vedadi M (2011). Thermal Denaturation Assays in Chemical Biology..

[10] Reed D, Shen Y, Shelat AA, Arnold LA, Ferreira AM (2010). Identification and characterization of the first small molecule inhibitor of MDMX.. J Biol Chem.

[11] Popowicz GM, Czarna A, Wolf S, Wang K, Wang W (2010). Structures of low molecular weight inhibitors bound to MDMX and MDM2 reveal new approaches for p53-MDMX/MDM2 antagonist drug discovery.. Cell Cycle.

[12] Pazgier M, Liu M, Zou G, Yuan W, Li C (2009). Structural basis for high-affinity peptide inhibition of p53 interactions with MDM2 and MDMX.. Proc Natl Acad Sci U S A.

[13] Bernal F, Wade M, Godes M, Davis TN, Whitehead DG (2010). A stapled p53 helix overcomes HDMX-mediated suppression of p53.. Cancer Cell.

[14] Bose P, Ozer H (2009). Neratinib: an oral, irreversible dual EGFR/HER2 inhibitor for breast and non-small cell lung cancer.. Expert Opin Investig Drugs.

[15] Kuhn DJ, Orlowski RZ, Bjorklund CC (2011). Second generation proteasome inhibitors: carfilzomib and immunoproteasome-specific inhibitors (IPSIs).. Curr Cancer Drug Targets.

[16] Ocana A, Amir E (2009). Irreversible pan-ErbB tyrosine kinase inhibitors and breast cancer: current status and future directions.. Cancer Treat Rev.

[17] Wallentin L (2009). P2Y(12) inhibitors: differences in properties and mechanisms of action and potential consequences for clinical use.. Eur Heart J.

[18] Feng BY, Shelat A, Doman TN, Guy RK, Shoichet BK (2005). High-throughput assays for promiscuous inhibitors.. Nature chemical biology.

[19] Arnold LA, Estebanez-Perpina E, Togashi M, Jouravel N, Shelat A (2005). Discovery of small molecule inhibitors of the interaction of the thyroid hormone receptor with transcriptional coregulators.. J Biol Chem.

[20] Vedadi M, Niesen FH, Allali-Hassani A, Fedorov OY, Finerty PJ (2006). Chemical screening methods to identify ligands that promote protein stability, protein crystallization, and structure determination.. Proc Natl Acad Sci U S A.

[21] Hwang JY, Attia RR, Zhu F, Yang L, Lemoff A (2012). Synthesis and Evaluation of Sulfonylnitrophenylthiazoles (SNPTs) as Thyroid Hormone Receptor-Coactivator Interaction Inhibitors..

[22] Hwang JY, Huang W, Arnold LA, Huang R, Attia RR (2011). Methylsulfonylnitrobenzoates, a new class of irreversible inhibitors of the interaction of the thyroid hormone receptor and its obligate coactivators that functionally antagonizes thyroid hormone.. J Biol Chem.

[23] Hwang JY, Arnold LA, Zhu F, Kosinski A, Mangano TJ (2009). Improvement of pharmacological properties of irreversible thyroid receptor coactivator binding inhibitors.. Journal of medicinal chemistry.

[24] Lavinder JJ, Hari SB, Sullivan BJ, Magliery TJ (2009). High-throughput thermal scanning: a general, rapid dye-binding thermal shift screen for protein engineering.. Journal of the American Chemical Society.

