# Supplementary figures and images for: On the Mechanism of Action of SJ-172550 in Inhibiting the Interaction of MDM4 and p53
Source: PLoS One. 2012 Jun 4;7(6):e37518. doi: 10.1371/journal.pone.0037518 (PMC3366986; doi:10.1371/journal.pone.0037518)

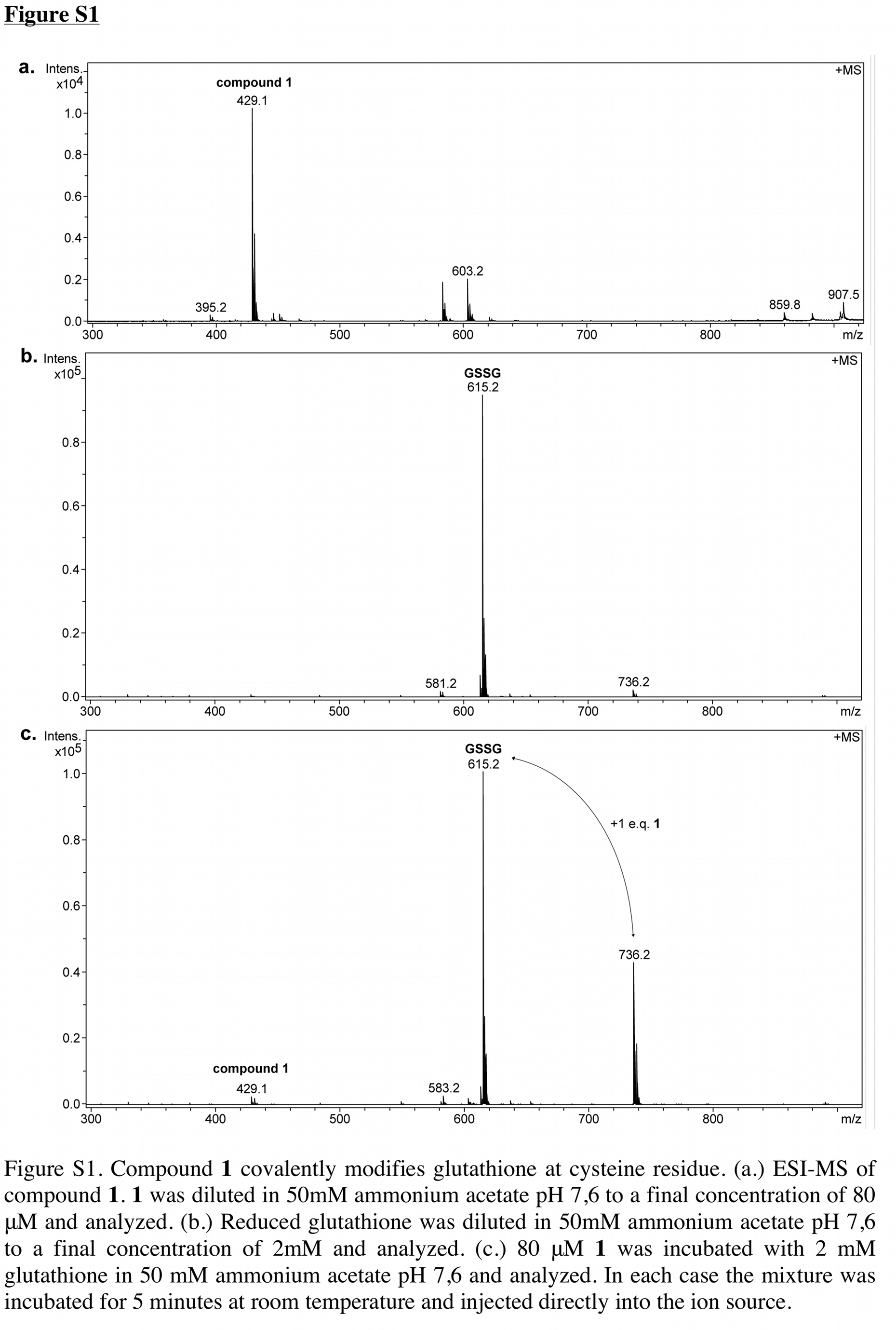

Supplement: Figure S1 — Formation of adducts by glutathione and compound 1. (TIF) [file pone.0037518.s001.tif]

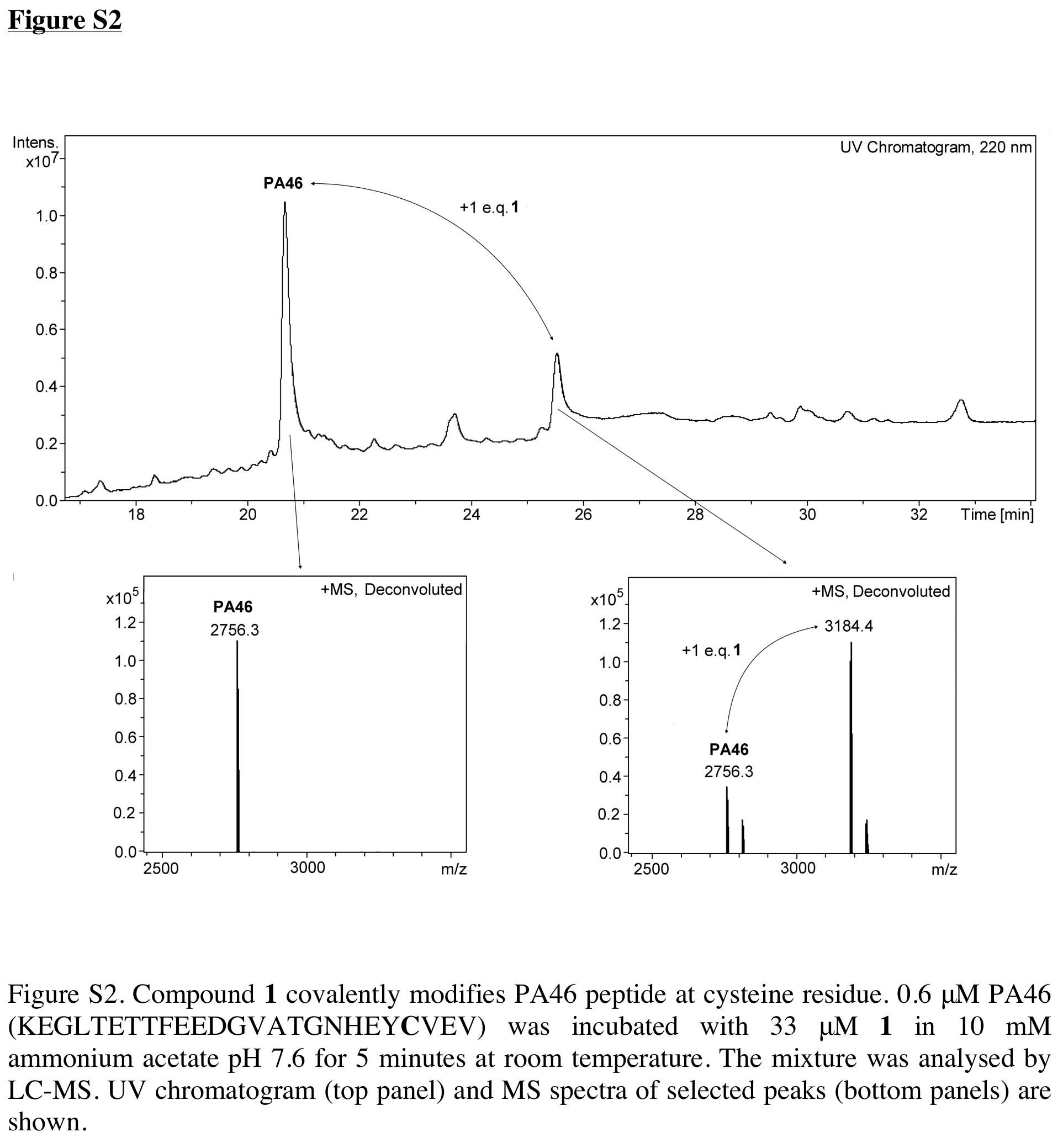

Supplement: Figure S2 — Formation of adducts by cysteine containing peptide and compound 1. (TIF) [file pone.0037518.s002.tif]

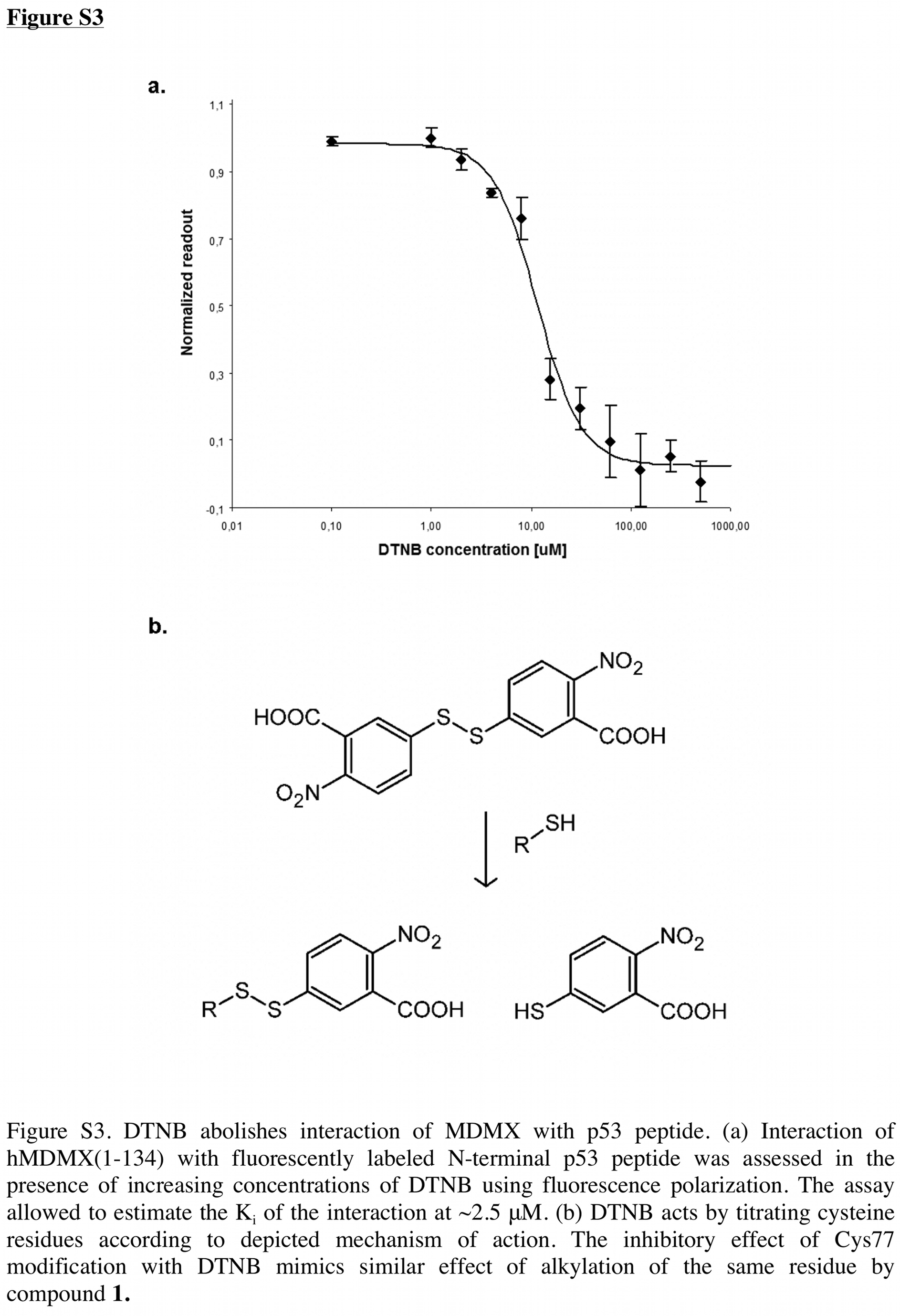

Supplement: Figure S3 — Inhibition of interaction of p53 and MDMX with DTNB. (TIF) [file pone.0037518.s003.tif]

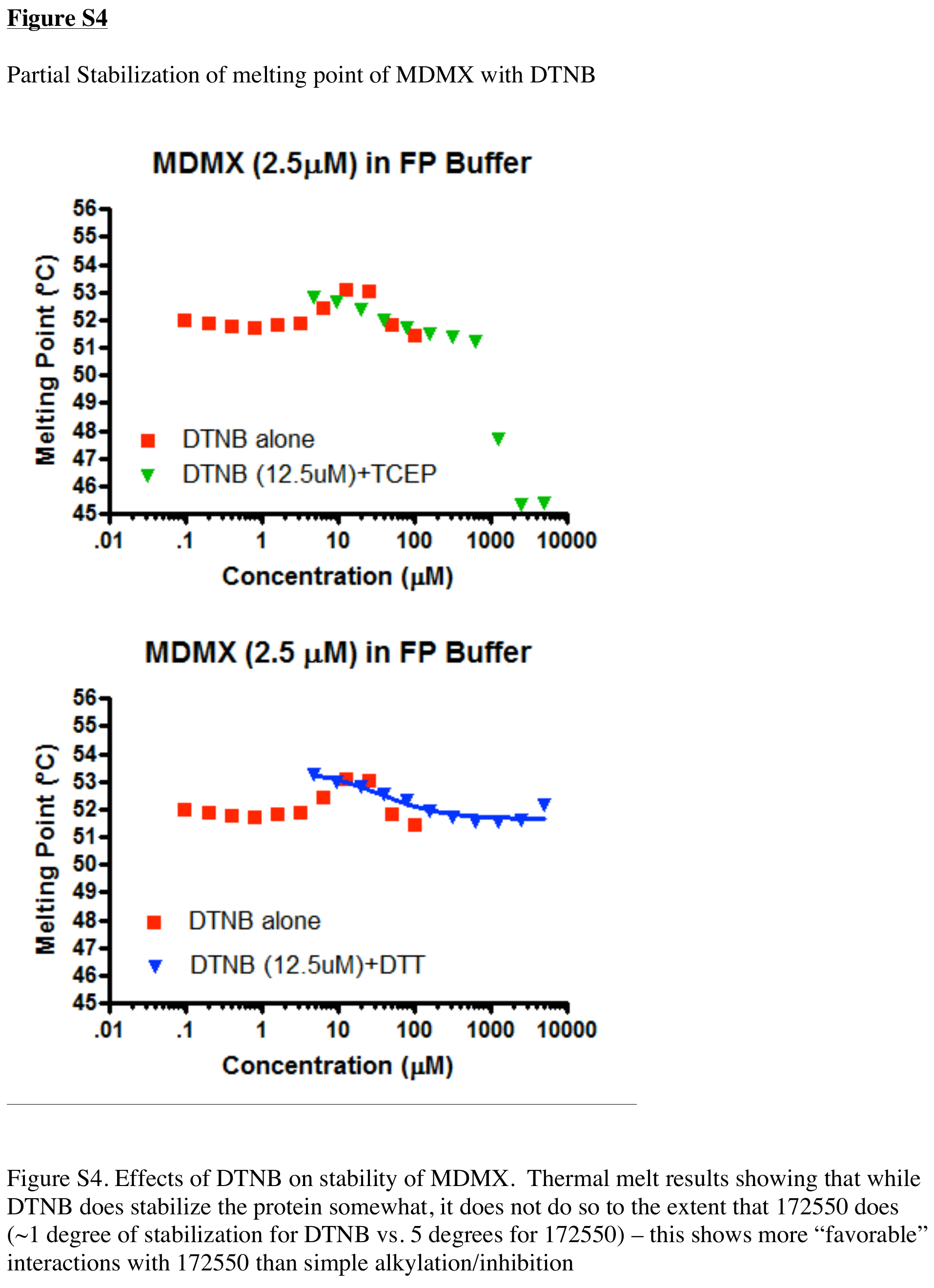

Supplement: Figure S4 — Partial Stabilization of melting point of MDMX with DTNB. (TIF) [file pone.0037518.s004.tif]

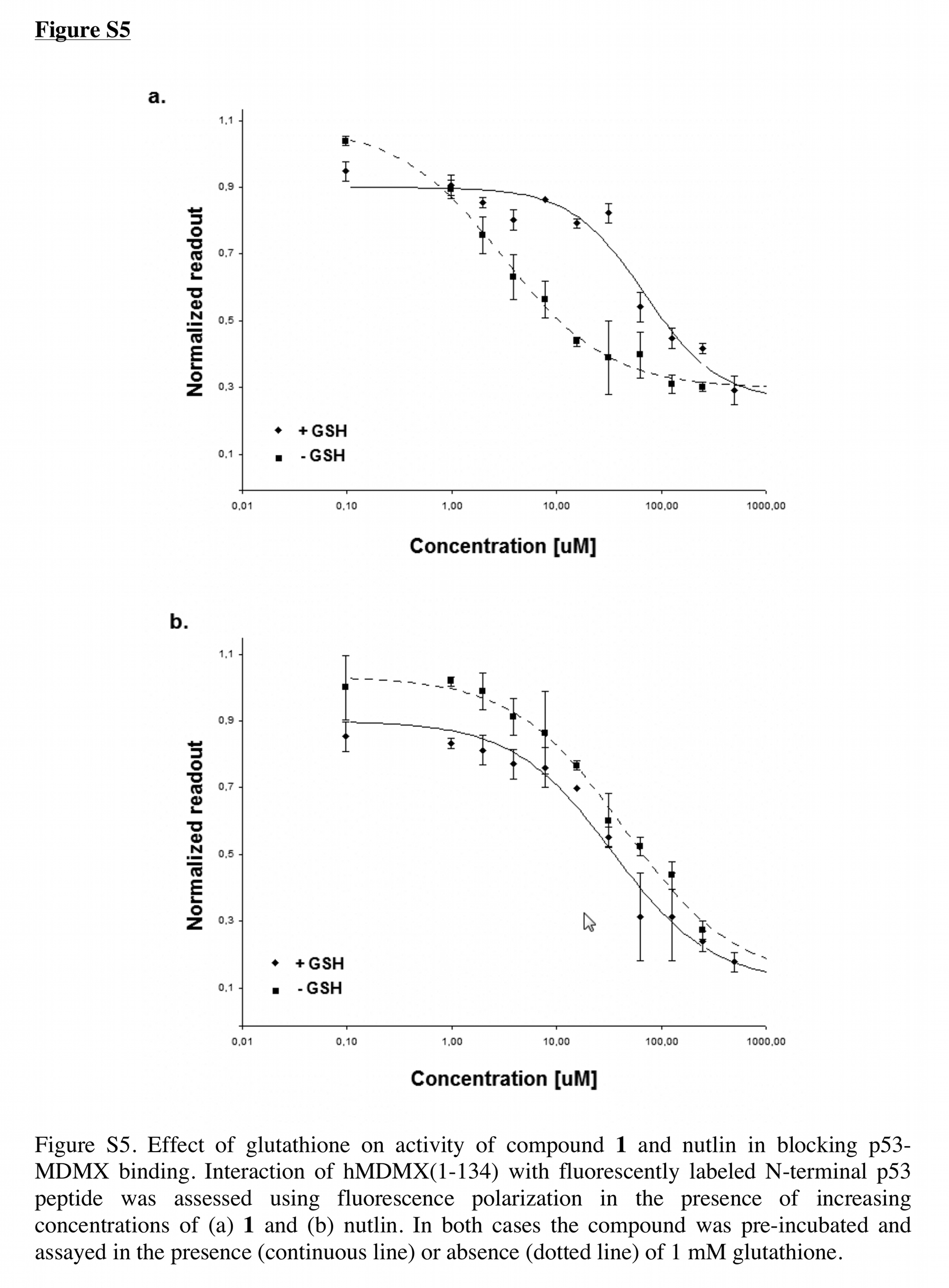

Supplement: Figure S5 — Effects of glutathione upon potency of compound 1 and nutlin in blocking binding of p53 peptide by MDMX. (TIF) [file pone.0037518.s005.tif]

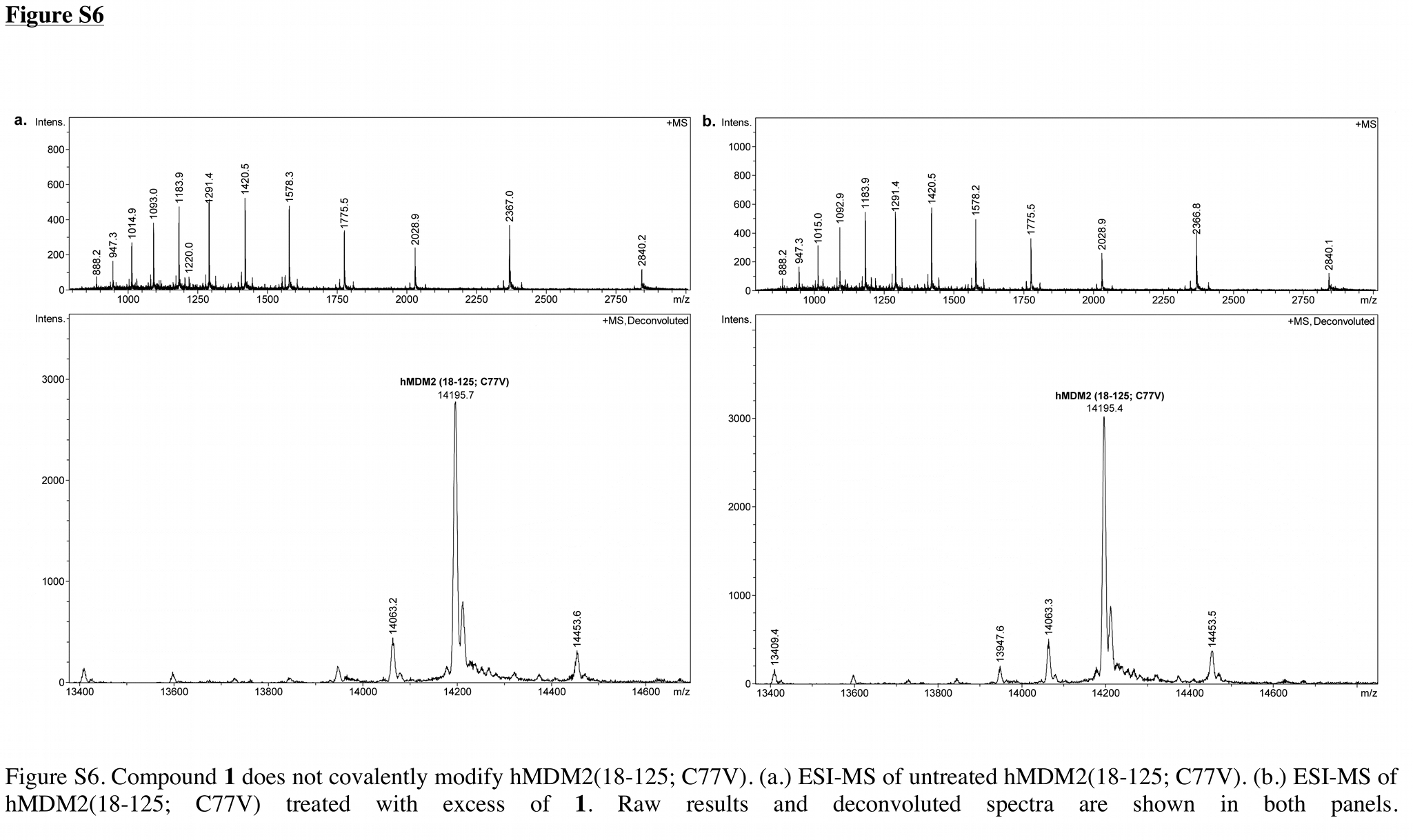

Supplement: Figure S6 — C77A mutation abolishes the ability of compound 1 to covalently label MDM2. (TIF) [file pone.0037518.s006.tif]

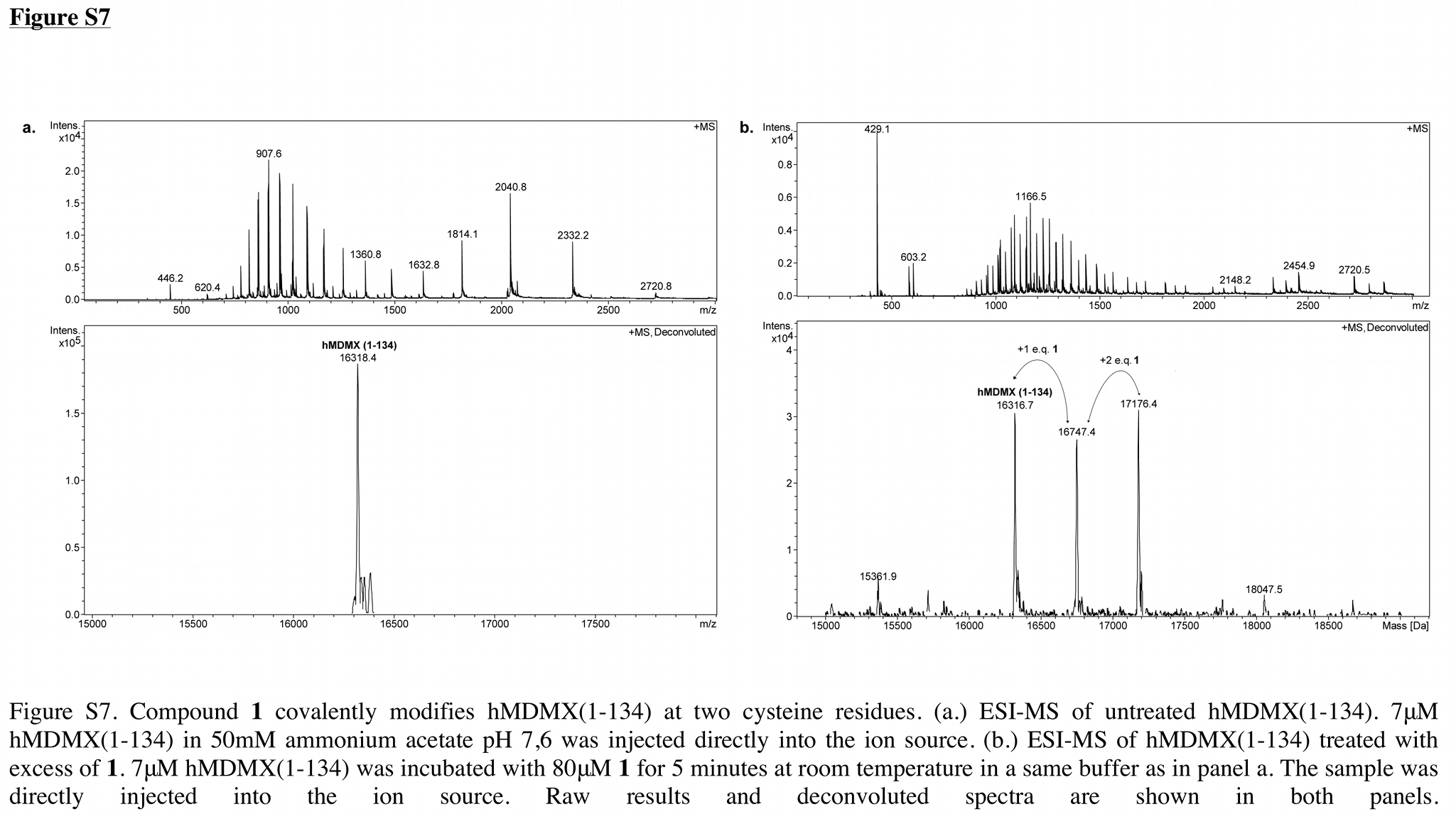

Supplement: Figure S7 — Formation of adducts by hMDMX(1–134) and compound 1. (TIF) [file pone.0037518.s007.tif]

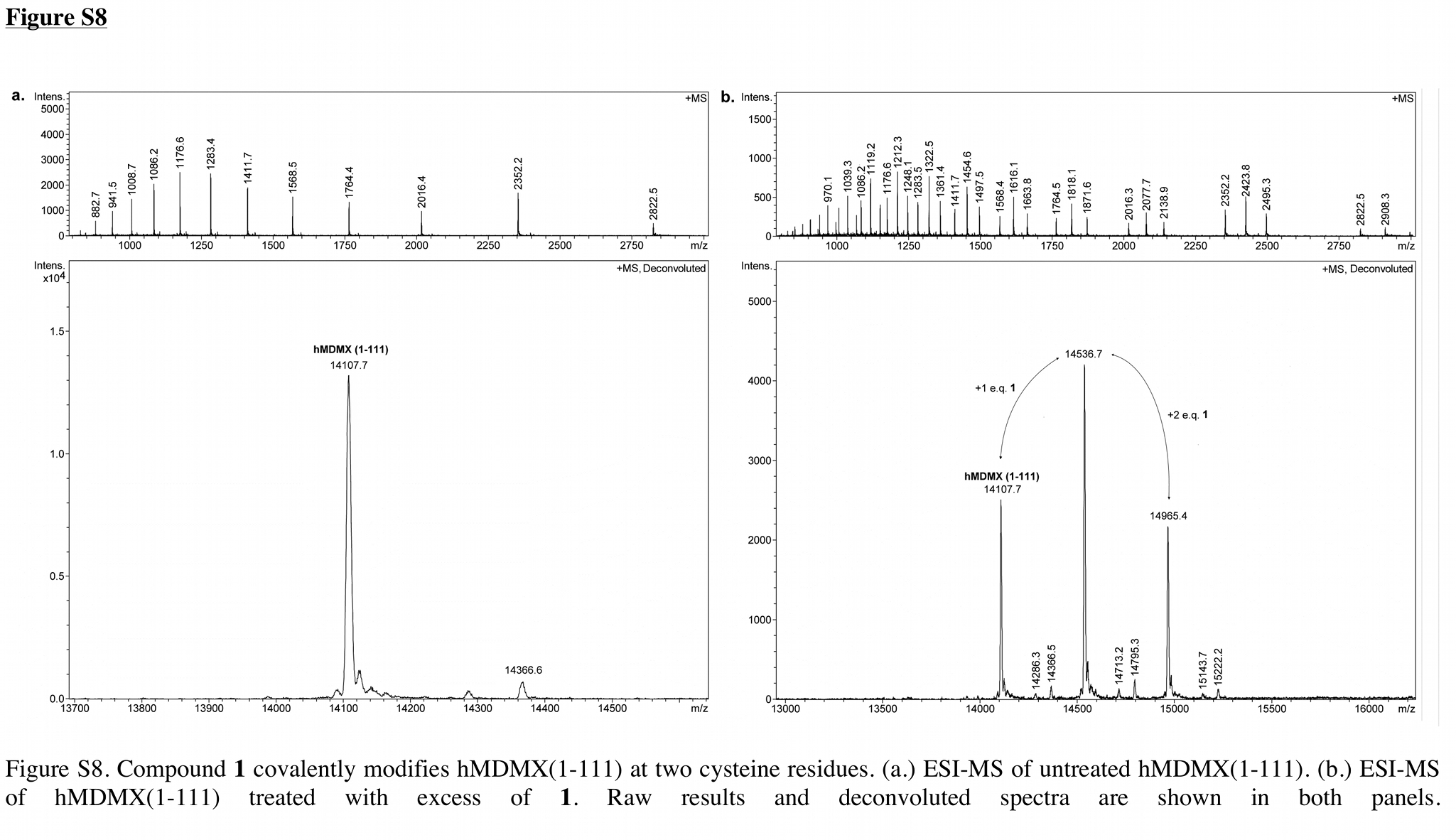

Supplement: Figure S8 — Formation of adducts by hMDMX(1–111) and compound 1. (TIF) [file pone.0037518.s008.tif]

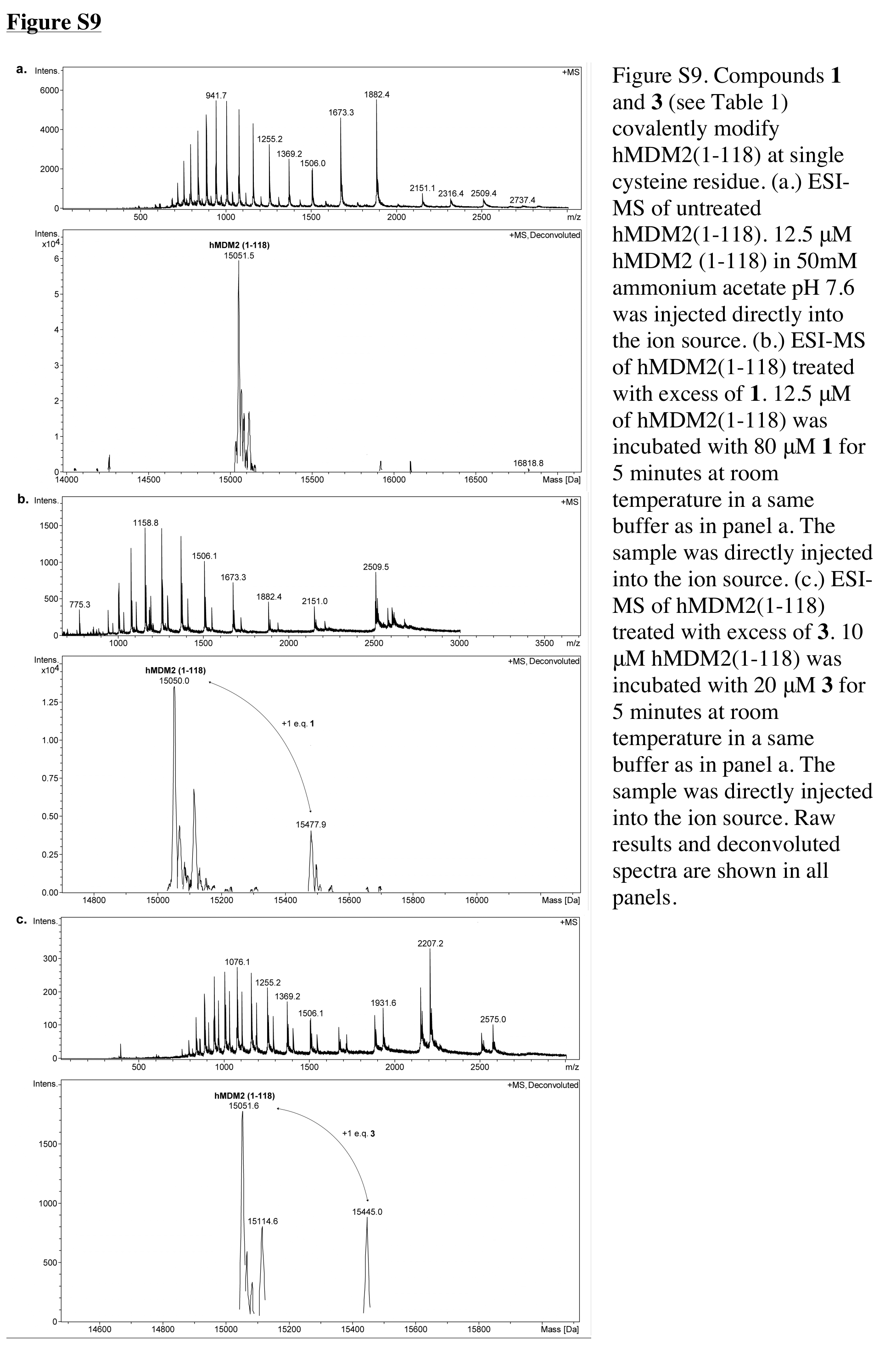

Supplement: Figure S9 — Formation of adducts by hMDM2(1–118) and compounds 1 and 3. (TIF) [file pone.0037518.s009.tif]

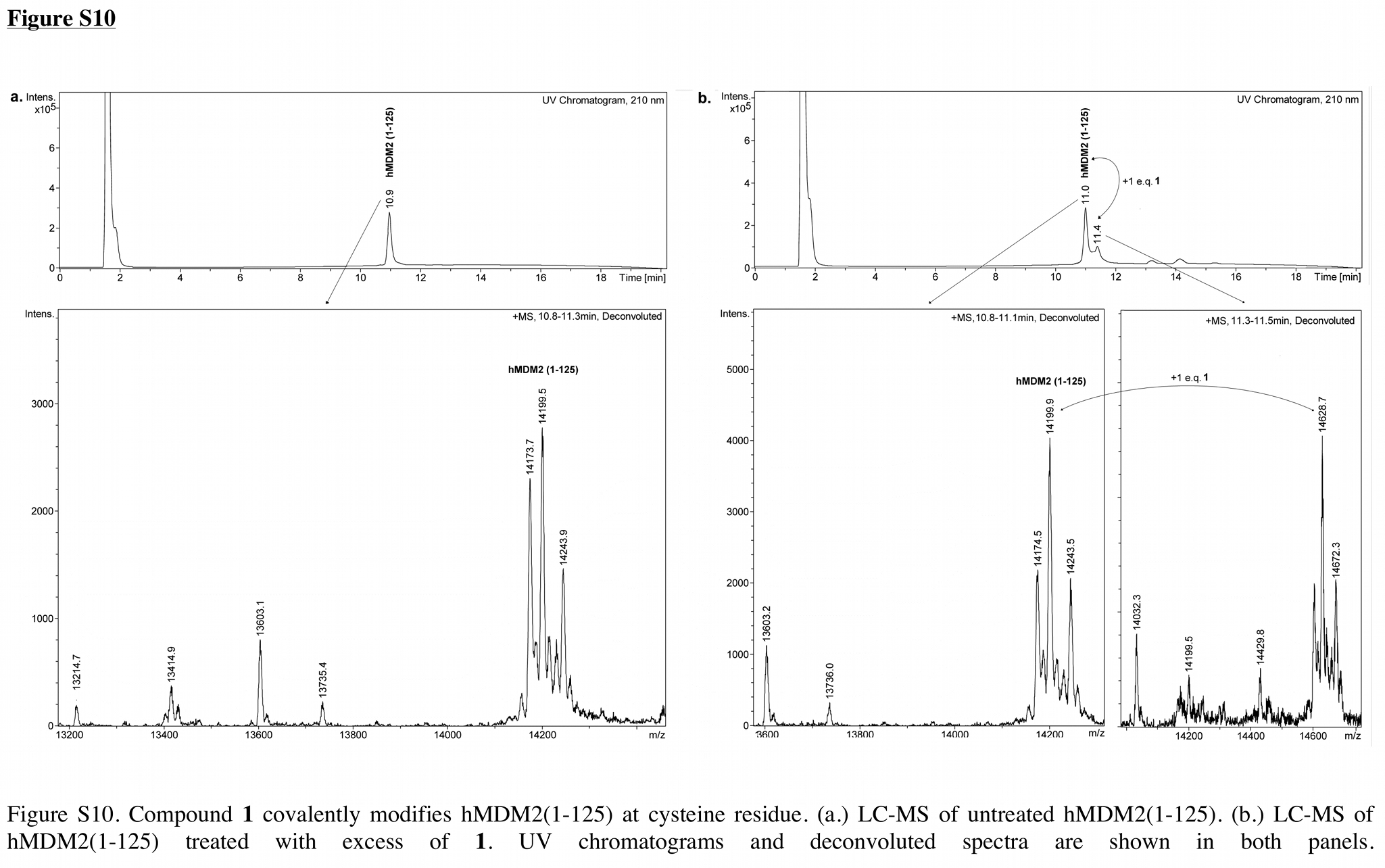

Supplement: Figure S10 — Formation of adducts by hMDM2(1–125) and compound 1. (TIF) [file pone.0037518.s010.tif]

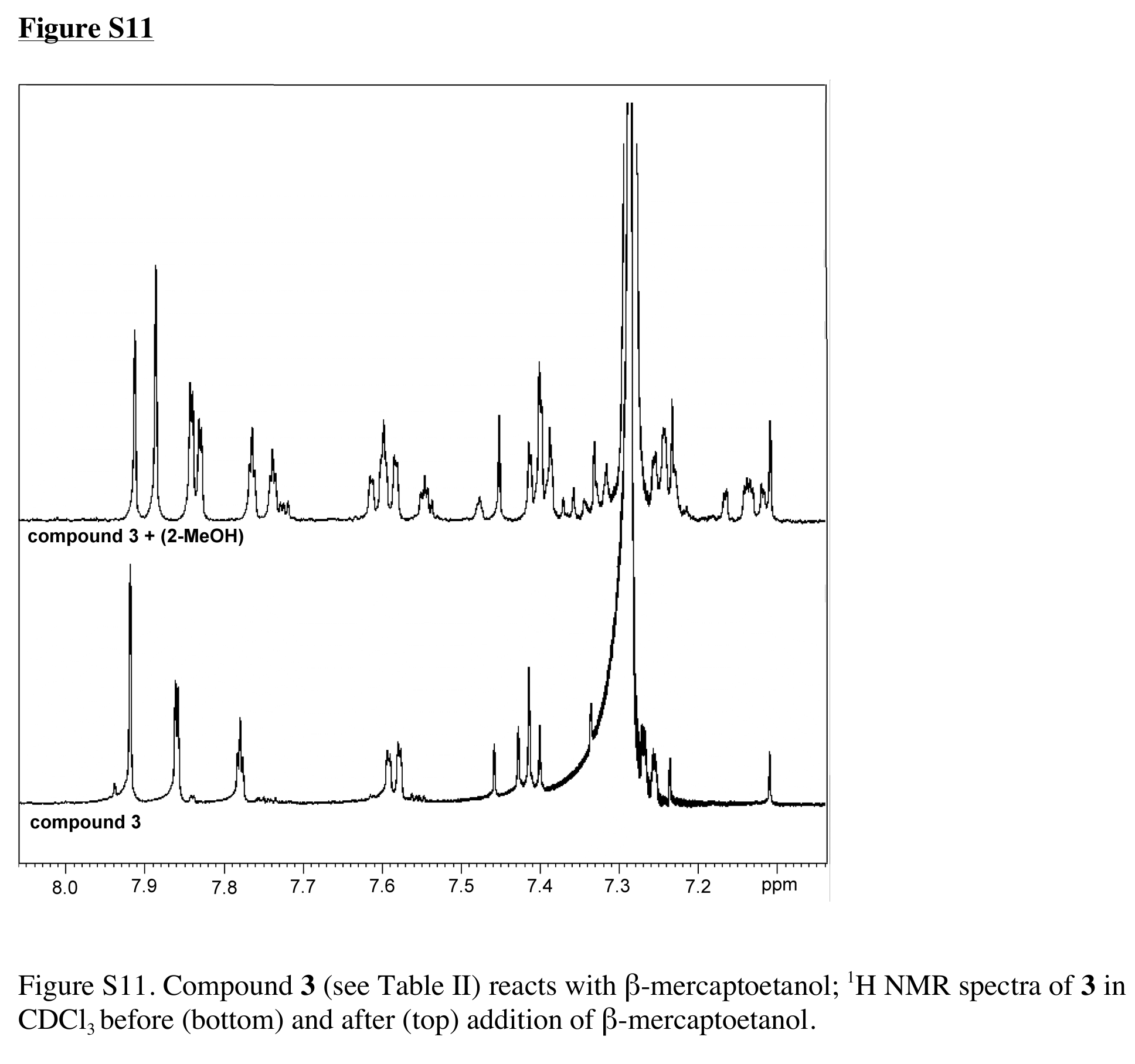

Supplement: Figure S11 — Formation of adducts by β-mercaptoetanol and compound 3 evidenced by NMR. (TIF) [file pone.0037518.s011.tif]

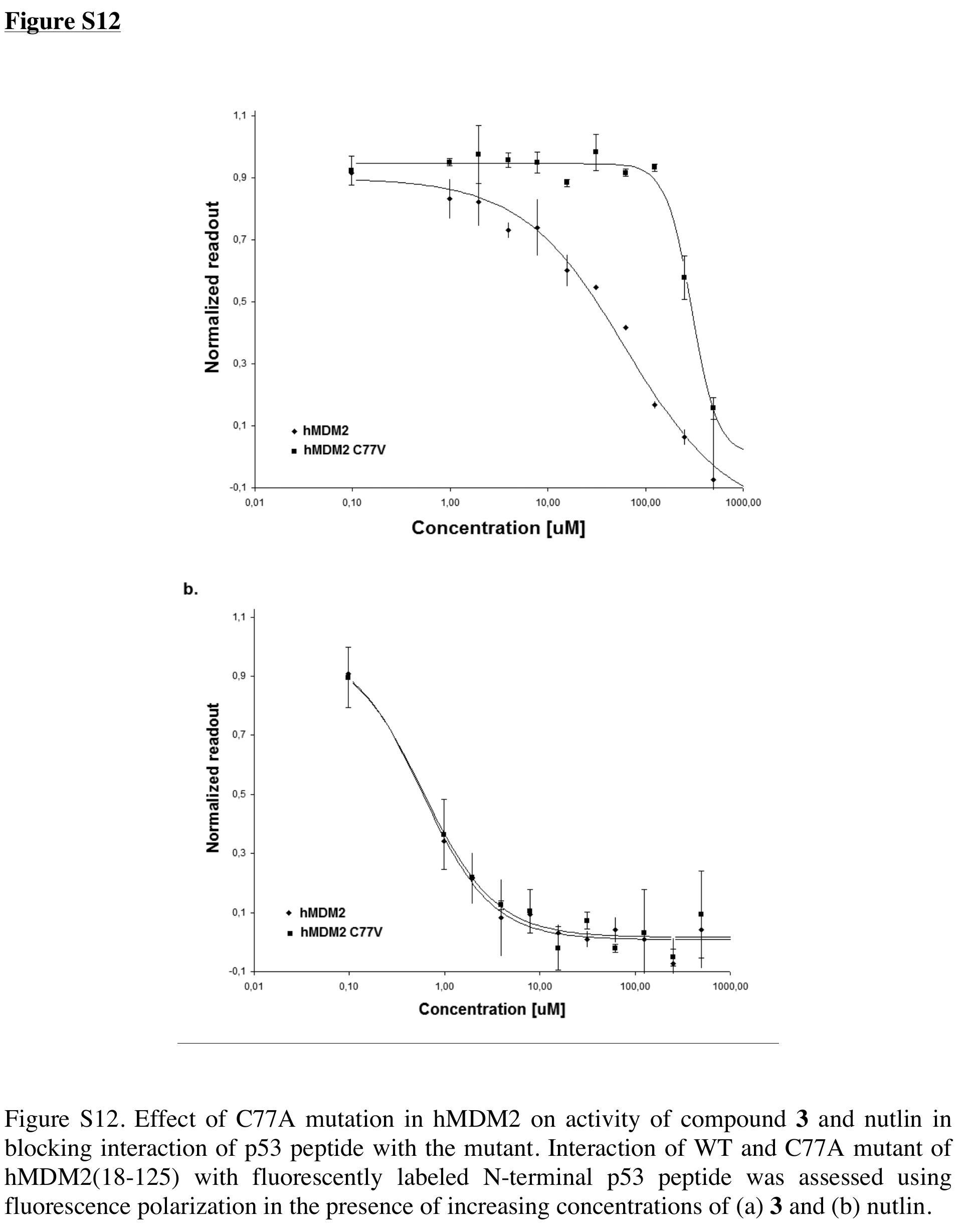

Supplement: Figure S12 — Reduced sensitivity of C77A mutant of MDM2 to compound 3 but not nutlin in blocking the interaction with p53 peptide. (TIF) [file pone.0037518.s012.tif]
