# Supplementary material for: On the Mechanism of Action of SJ-172550 in Inhibiting the Interaction of MDM4 and p53
Source: PLoS One. 2012 Jun 4;7(6):e37518. doi: 10.1371/journal.pone.0037518 (PMC3366986; doi:10.1371/journal.pone.0037518)
Supplement: Table S1 — Binding affinities of close and distant derivatives of 1 towards Mdm2 and Mdmx. (PDF) [file pone.0037518.s013.pdf]

Table SI

Affinity of compounds containing the chemotype of **1** or comparable chemotype containing the α,β-unsaturated amide towards Mdm2 and Mdmx as determined using fluorescence polarization p53 peptide exclusion assay. Compound core according to J. Biol. Chem. 2010; *285*: 10786 is marked in blue. ND – compound does not noticeably block interaction with p53 peptide.

Compound core (J. Biol. Chem. 2010; *285*: 10786)	Chemical formula	Compound code	Mdm2 Ki [uM]	MdmX Ki [uM]
cluster 1	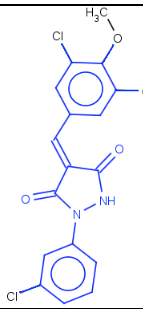	2K01 (compound **3**)	1.34	1.76
cluster 1	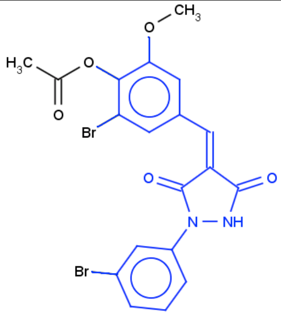	4K67	5.04	0.99
cluster 4	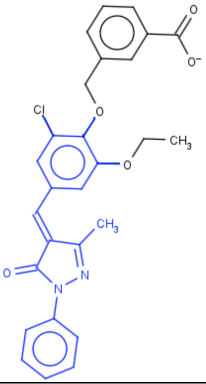	8X50	20.51	1.09
cluster 4	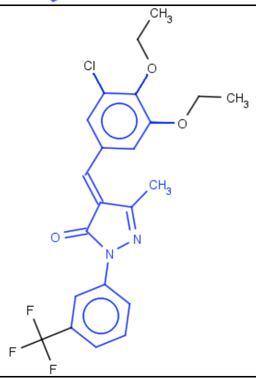	4M10	ND	1.6

cluster 4	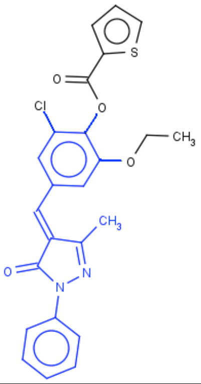	7L41	ND	2.35
cluster 4	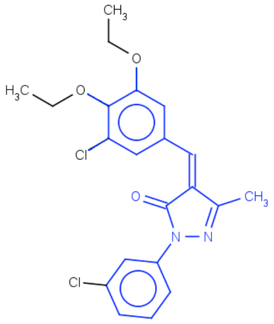	1L81	ND	2.46
cluster 4	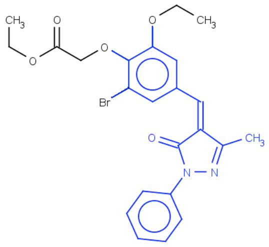	4R16	2.57	5.25
cluster 4	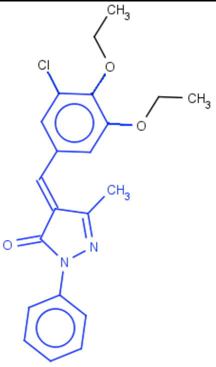	8X14	29.99	6.66
cluster 4	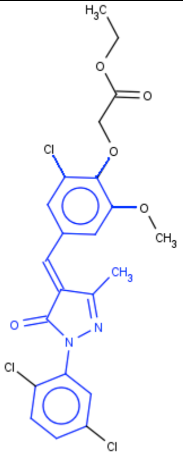	1W18	ND	7.24

cluster 4	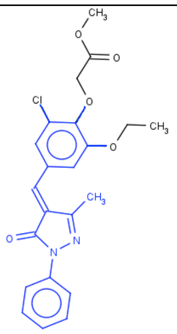	4T14 (compound **1**) (SJ-172550)	5.21	8.07
cluster 4	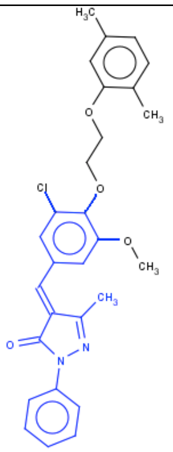	9S01	ND	17.73
cluster 4	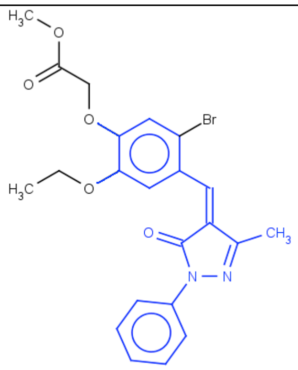	0M61	33.8	36.47
cluster 4	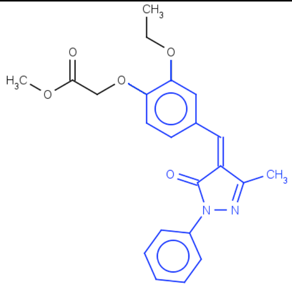	2T49	41.65	47.12

cluster 4	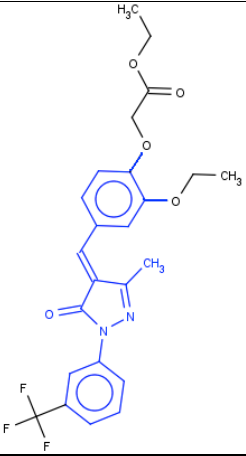	5S34	ND	48.86
cluster 4	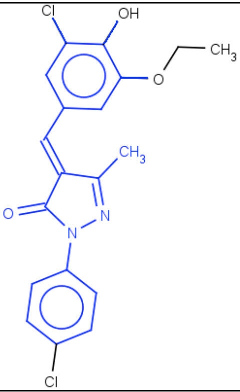	1W63	43.53	117.082
cluster 4	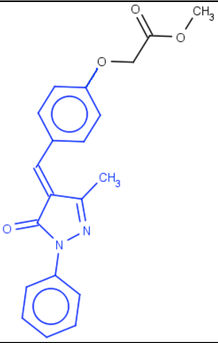	7U61	ND	163.97
cluster 4	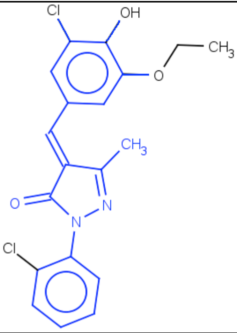	2K38	ND	ND
